# Memory CD73^+^IgM^+^ B cells protect against *Plasmodium yoelii* infection and express Granzyme B

**DOI:** 10.1371/journal.pone.0238493

**Published:** 2020-09-04

**Authors:** Marcela Parra, Megan Weitner, Amy Yang, Adovi Akue, Xia Liu, Thomas Schmidt, Windy R. Allman, Mustafa Akkoyunlu, Steven C. Derrick

**Affiliations:** United States Food and Drug Administration, Center for Biologics and Research, Division of Bacterial, Parasitic and Allergenic Diseases, Silver Spring, Maryland, United States of America; Agency for Science, Technology and Research - Singapore Immunology Network, SINGAPORE

## Abstract

To better understand anti-malaria protective immune responses, we examined the cellular mechanisms that govern protective immunity in a murine *Plasmodium yoelii* 17X NL (PyNL) re-infection model. Initially, we confirmed that immune B cells generated during a primary PyNL infection were largely responsible for protection from a second PyNL infection. Using the previously identified memory B cell markers CD80, PD-L2, and CD73, we found an increase in the frequency of CD80^-^PD-L2^-^CD73^+^ B cells up to 55 days after a primary PyNL infection and at 4–6 days following a second PyNL infection. Moreover, injection of enriched immune CD19^+^CD73^+^ B cells into nonimmune mice were significantly more protective against a PyNL infection than CD73^-^ B cells. Interestingly, a substantial fraction of these CD73^+^ B cells also expressed IgM and granzyme B, a biomolecule that has been increasingly associated with protective responses against malaria.

## Introduction

Globally, malaria remains one of the most widespread and deadly human infectious diseases. Despite successful interventions such as bed nets, an estimated 228 million cases of malaria and 405,000 malaria-related deaths occurred in 2018 [1]. Most of these occurred in children living in Africa where a child dies every 5 minutes from a malaria infection. Success in developing vaccines has been limited largely because protective immunity to malaria is complex and generally not well understood. Seminal studies showing that passive transfer of antibodies from clinically immune adults to actively infected children reduced disease symptoms demonstrated a critical role of the humoral response in preventing malarial disease [2]. Moreover, humoral responses have been shown to play an important role in naturally acquired immunity to malaria, but the protective responses usually develop slowly in humans and are often difficult to maintain [3]. Studies in animal models, including the PyNL mouse model of malaria, have confirmed the importance of anti-malarial humoral immunity and, accordingly, have identified B cells as critical components of protection against malaria [4–8]. The PyNL strain causes a self-resolving murine infection which clears in about 3 weeks. Mice that recover from the primary infection have prolonged immunity to reinfection, and the passive transfer of immune spleen B cells or hyper-immune sera have been shown to generate protection against a PyNL infection [5].

The prolonged protection conferred by a primary PyNL infection strongly suggests that malaria-specific memory B cells (MBC) are efficiently generated after a single infection [9, 10]. Overall, activation of pathogen-experienced MBC is a critical component of the host defense against reinfection with different pathogens. Typically, an efficient MBC recall response can be initiated during a pathogen re-exposure through the recognition of antigens resulting in MBC proliferation [11]. The secondary MBC response usually produces elevated concentrations of antibodies with higher affinities than the primary humoral response. However, an incomplete understanding of functional and phenotypic characterizations of murine MBC has limited the investigation of murine B cell memory in malaria resistance. Consequently, despite the critical importance of humoral immunity in protecting against re-infection with the malaria parasite as well as infection with other parasites, the biological nature of mouse anti-parasitic MBC responses are exceedingly complex with new insights into the mechanisms of protection recently coming to light.

For many pathogens, B cell memory responses are complex and distinct classes of MBC develop (9). Work by Shlomchik and colleagues described five diverse MBC subsets based on staining for markers CD80, PD-L2 and CD73 [12, 13]. These MBC subset diversities were independent of class switching because IgM and IgG bearing MBC were found in each phenotypic group. Furthermore, the distinct MBC subsets have properties that showed progression from naive-like to more memory-like cells. Studies in mice immunized with a cholera toxin adjuvanted preparation or re-infected with influenza virus have provided validation for the use of these memory B cell markers [14, 15]. These markers can be used to advance our understanding of the production and maintenance of anti-malarial MBC, and in the development of vaccines against malaria.

Here, we have characterized immune B cell subsets in PyNL infected mice using the CD80, PD-L2, and CD73 surface markers. We showed that the protective responses induced by a primary infection were primarily mediated by CD73^+^ B cells. Interestingly, most of the immune CD73^+^ B cells also expressed IgM, and a significant fraction expressed granzyme B (GrB).

## Materials and methods

### Mice

C57BL/6 female mice 6 to 10 weeks of age were purchased from the Jackson Laboratories (Bar Harbor, ME). This study was done under the guidance for the care and use of laboratory animals specified by the National Institutes of Health. Euthanasia was administered via cervical dislocation. All experimental procedures were approved by the Institutional Animal Care and Use Committee of the Center for Biologics Evaluation and Research under Animal Study Protocol 2008–08.

### Parasites

PyNL infected mouse red blood cells (RBC) were stored as a frozen stock and used to infect C57BL/6 mice by the intraperitoneal route (i.p.). When approximately 10% parasitemia levels were observed by examining Giemsa blood smears, blood was collected and diluted in PBS to achieve a concentration of 1 x 10^6^ parasites/200 μl to infect mice. Percent parasitemia (parasitized RBCs/total RBCs x 100) was measured starting at day 3–5 post-infection and until parasitemia clearance. PyNL infected red blood cells were also used at a concentration of 5 x 10^6^/500 μl/well to study *in vitro* recall responses.

### Adoptive transfer of B cells

Spleen cells from naive C57BL/6 mice and from PyNL immune mice that had cleared infection for 9 months were isolated and treated with ACK lysing buffer (Lonza, Rockville, MD) to remove erythrocytes. For B cell enrichment, splenocytes were incubated with anti-CD19 antibody conjugated to microbeads per manufacturer’s instructions (Miltenyi Biotec; San Diego, CA), and then passed through a magnetic column (LS column, Miltenyi Biotec). The bound CD19^+^ cells were collected by removing the column from the magnet and flushing the cells into a tube using PBS-2% FBS. The purity of CD19^+^ B cells was assessed by flow cytometry and was found to be >95%. Enriched PyNL immune B cells were then adoptively transferred by intravenous (i.v.) injection of 3 x 10^7^ immune cells per mouse. Two hours after B cell transfer, mice were infected with 1 x 10^6^ PyNL infected RBC. The percent parasitemia after infection was determined by examining Giemsa-stained thin blood smears.

### Adoptive transfer of CD73^+^ and CD73^-^ B cells

Spleen cells from C57BL/6 mice that had cleared a PyNL infection 7 months earlier were isolated and treated with ACK lysing buffer to remove erythrocytes. Splenocytes were depleted of Thy1.2^+^ cells using anti-Thy1.2 antibody coated microbeads (Miltenyi Biotec), and the cells that passed through a magnetic column (Thy1.2 negative) were collected and washed. The unbound, enriched B cells were incubated with Fc block antibody, and the CD73^+^ B cells were enriched using biotinylated anti-CD73 antibody followed by washing and incubation with anti-biotin microbeads. The CD73^-^ cells passed through the column and were collected separately from the CD73^+^ cells which bound to the column and were recovered by removing the column from the magnet and flushing the cells from the column using PBS/FBS. PyNL immune CD73^+^ and CD73^-^ B cells were then adoptively transferred by i.v. injection of 9 x 10^6^ or 3 x 10^7^ immune cells per mouse. Two hours following the B cell transfer, mice were injected with 1 x 10^6^ PyNL infected RBC. The percent parasitemia after infection was determined by examining Giemsa-stained thin blood smears.

### *In vitro* studies

Spleens were aseptically removed from naive and PyNL immune mice (n = 5) 4 months post-primary PyNL infection and homogenized to obtain a single cell suspension. Cell suspensions from individual mice were treated with ACK lysis buffer to deplete erythrocytes (5 min at room temperature), washed with cold RPMI and then re-suspended at a concentration of 5 x 10^6^ spleen cells/ml with 5 x 10^6^ PyNL infected erythrocytes in RPMI media (10% Fetal Bovine Serum, 1% NEAA, 1% Glutamine; 0.01 μM 2- mercaptoethanol). Cell cultures were incubated at 37°C (5% CO_2_) in a 1.0 ml volume per well of a 24-well plate. Control wells contained 5 x 10^6^ spleen cells without PyNL infected RBCs. Following culture for 4 days the cells were harvested and prepared for conventional flow cytometry analysis and flow cytometry imaging as described below. Supernatants were harvested and used for GrB measurements by ELISA (R&D Biosystems, Minneapolis MN).

### Flow cytometry

Flow cytometry was used to measure the frequencies of splenic B cells expressing CD73, GrB, and IgM surface markers during the course of a primary PyNL infection (days 0, 6, 13 and 22), 1 month post-parasitemia clearance (1-M PC), 1 day before secondary infection (Pre-SI), and 6 days post-secondary infection (D-6 Post-SI). At each time point, a single cell suspension of splenocytes was incubated with Near-IR live-dead stain (Molecular Probes; Eugene, Oregon) and Fc block for 15 min followed by staining with antibodies specific for CD19 (FITC; clone eBio1D3) (eBioscience, Waltham, MA), PD-L2 (Biotin; clone Ty25) (BioLegend, San Diego, CA), CD80 (PE; clone 16-10A1) (BioLegend), CD73 (APC, clone TY/11.8) (Miltenyi Biotec), CD159 (Percp-Cy5.5; clone 20d5) (BD Pharmingen, Franklin Lakes, NJ), or IgM (PE-Cy7; clone R6-60.2) (BD Pharmingen) (0.2 μg each) molecules in a 50–100 μl volume. Following a 30-min incubation at 4°C, the cells were washed twice with PBS + 2% FBS (FACS buffer) and fixed with 2% paraformaldehyde for 30 min. For detection of biotin-conjugated antibodies, the cells were incubated with Qdot605, streptavidin conjugate (1 μl) (Molecular Probes) in a volume of 0.1 ml for 15 min and then washed prior to fixing the cells. For intracellular staining, the fixed cells were permeabilized by washing the cells twice with Perm/Wash buffer (0.1% saponin, 1.0% FBS, 1.0% HEPES in PBS) followed with the addition of antibodies specific for GrB (PE; clone NGZB) (eBioscience) (0.25μg) and IL-10 (APC; clone JES-16E3) (eBioscience) (0.25 μg) in a 50–100 μl vol. After incubating the cells for 45 min at room temperature, the cells were washed twice with Perm/Wash buffer followed with a wash with FACS buffer. The frequency of cells expressing each antigen was determined by flow cytometry (LSR II; BD Biosciences) and FlowJo software (Ashland, OR). Results are presented as frequencies and/or total number of cells per 1 x 10^6^ B cells.

### Flow cytometry imaging

To visualize CD19^+^ cells expressing CD73 and GrB from the *in vitro* experiment, an ImageStreamX MKII Analyzer (Luminex Corp.; Austin, TX) was used. Following a 4 day incubation with iRBCs, splenocytes were labeled with violet live-dead stain to differentiate viable from dead cells and then surface stained using antibodies with the following fluorochromes and specificities: FITC-CD19 (clone MB19-1), BV420-CD8 (clone 53–6.7) (Biolegend), APC-CD73 (clone TY/11.8) (Biolegend), and PE-Granzyme B (clone NGZB) as described above. Following fixation, the cells were visualized using the ImageStreamX MKII analyzer and the acquisition software INSPIRE. After gating on live CD19^+^ cells, a gate was placed on CD73, GrB double-positive cells to visualize via bright field or fluorescence mode at 60X magnification. Staining with antibody specific for CD8 ensured that the cells being gated on and visualized were not CD8^+^.

### Statistical analyses

Graph Pad Prism 7 software was used to analyze the data for these experiments (Graph Pad Software, San Diego, CA). Statistical analysis of parasitemia, flow cytometry and GrB data were evaluated using the Mann-Whitney test. The antibody ELISA data were evaluated using the Ordinary One-way ANOVA test. Variability is expressed as the standard error of the mean (± SEM) throughout the paper.

## Results

### PyNL immune B cells confer protection against a second PyNL infection

**Fig 1:**
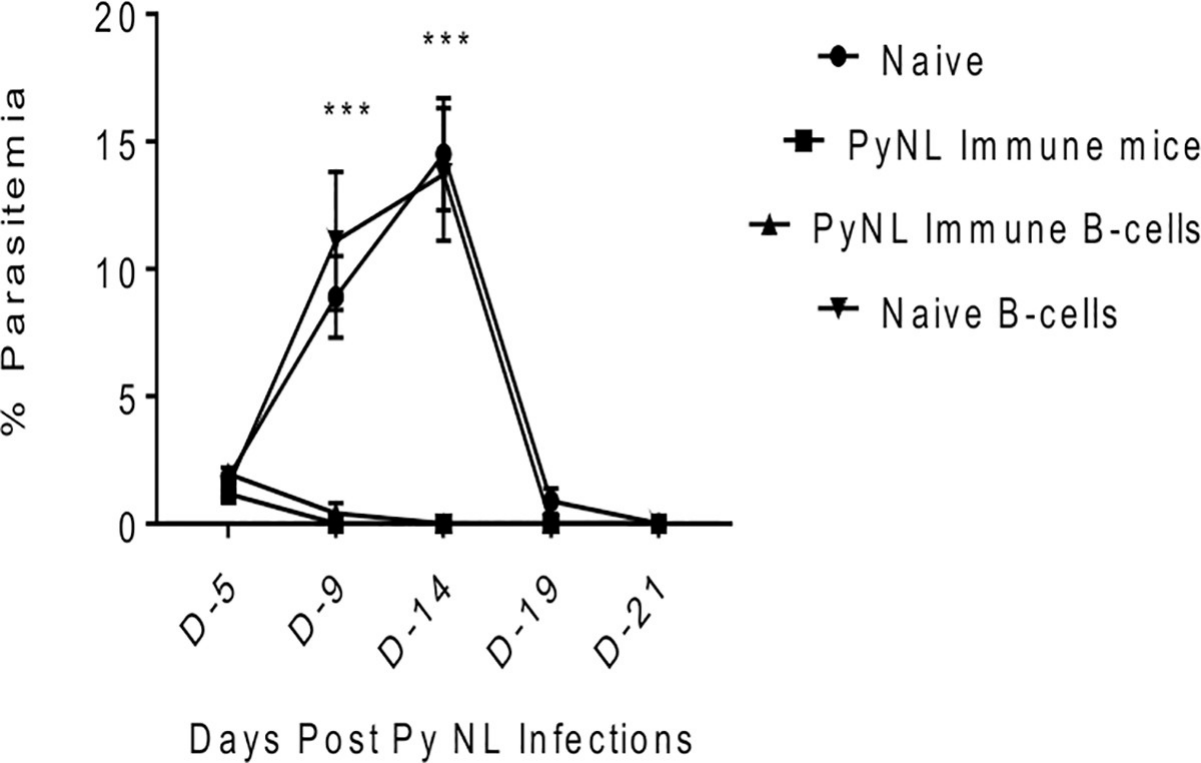
B cells enriched from splenocytes 9 months post-PyNL clearance conferred protection against a secondary PyNL infection. Results are expressed as percent parasitemia ± SEM following i.p. infection of C57BL/6 mice with 1 x 10^6^ PyNL erythrocytic stage parasites. Parasitemia levels from naive (nonimmune), PyNL Immune mice and mice adoptively transferred with B cells from naive mice were compared with mice receiving PyNL immune B cells. Parasitemia levels were evaluated by examining blood smears beginning at day 5 post-infection until parasitemia clearance. Mann-Whitney test was used for statistical evaluation; *p<0.05; n = 7 mice per group.

Infection with PyNL parasites renders mice resistant to a second PyNL infection but the mechanisms of anti-parasitic protection are only gradually coming to light [16]. To delineate the cellular immune response involved in the protection of mice to a second PyNL infection, we injected 1 x 10^6^ erythrocytic parasites to C57BL/6 mice 9 months after the clearance of the first PyNL infection. Concurrently, naive control mice were infected with the same dose of PyNL infected erythrocytes. As seen in Fig 1, the malaria infection of naive mice followed a typical course with a peak parasitemia of 14.5 ± 2.2% seen at day 14 and clearance of the infection by day 21. In contrast, a second challenge of the PyNL experienced mice yielded an abortive infection with a maximal 1.2 ± 0.09% peak parasitemia detected at day 5 and complete parasite clearance observed by day 9. To confirm the role of B cells in protection against *Plasmodium* infections, B cells purified from the splenocytes of mice that had been infected with PyNL 9 months earlier were adoptively transferred into nonimmune animals. Splenic B cells harvested from naive mice or mice recovered from PyNL infection served as controls. As seen in Fig 1, the levels of parasitemia in mice that received B cells from naive (nonimmune) mice mirrored levels seen in naive mice with a peak parasitemia of 13.7 ± 2.5% detected at day 14. However, similar to results observed in immune mice, the parasitemia percentages and the time to clearance were significantly reduced relative to controls in mice adoptively transferred with immune B cells. These results confirm the role parasite experienced B cells play in resistance to PyNL infection.

### Expression of B cell surface markers during a primary PyNL infection

**Fig 2:**
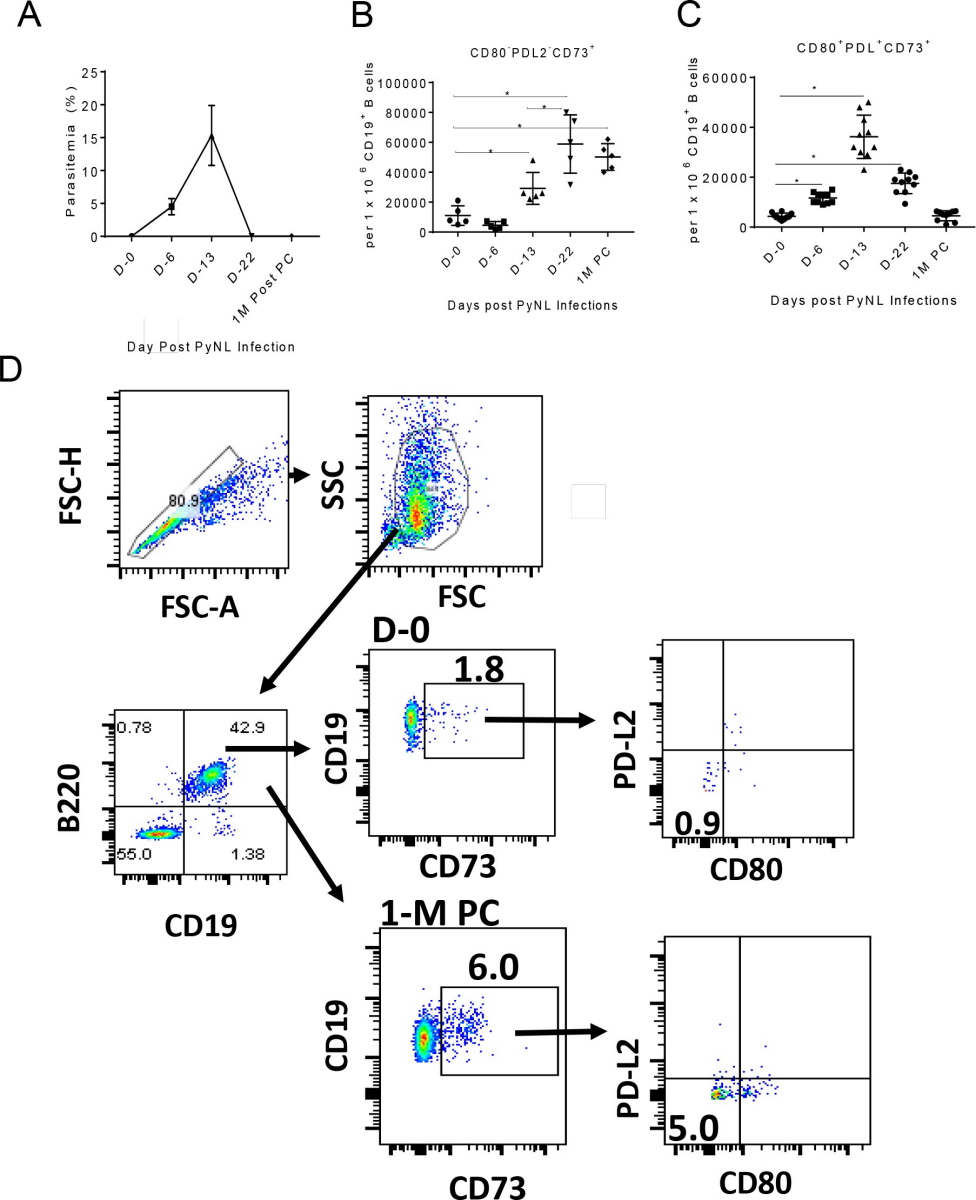
Kinetics of the expansion of CD19^+^ B cells expressing CD80, PD-L2 and CD73 markers post-primary PyNL infection. (A) Percent parasitemia over the course of a PyNL infection measured by blood smear evaluation. Expansion of splenic (B) CD80^-^PD-L2^-^CD73^+^ and (C) CD80^+^PD-L2^+^CD73^+^ B cell populations (mean ± SEM) measured by flow cytometry over the course of a PyNL infection and at 1 month post-parasitemia clearance (1-M PC). (D) Representative dot plots show gating strategy and frequencies of splenic CD80^-^PD-L2^-^CD73^+^ B cells from mice at D-0 and at 1-M PC. Mann-Whitney test was used for statistical evaluation; *p<0.05; n = 5 mice per group. Results are expressed as per 1 x 10^6^ CD19^+^ B cells from one experiment out of two with similar results. *p<0.05; n = 5 mice per group.

Uncertainty about the surface markers that define murine memory B cells has impeded the study of these cells in mice. Shlomchik and colleagues have described heterogeneous populations of nitro-phenyl reactive memory B cells that expressed CD80, PD-L2 and CD73 cell markers [9–12, 17]. These markers were also studied in mouse malaria models and have been shown to be expressed at different combinations following *P*. *chabaudi* infection [18]. To assess whether these B cell memory markers were relevant to immunity against PyNL infection, we initially evaluated the temporal kinetics of the expression of the CD80, PD-L2 and CD73 markers on CD19^+^ B cells during a primary PyNL infection. Fig 2A shows percent parasitemia over the course of a PyNL infection, with clearance by D-22 post-PyNL infection. Using flow cytometry to analyze splenocytes during the course of a PyNL infection at the time points corresponding to Fig 2A, we observe a significant increase in CD80^-^PD-L2^-^CD73^+^ B cell frequency over the course of infection with its peak at parasitemia clearance (D-22) (Fig 2B). The number of CD80^-^PD-L2^-^CD73^+^ B cells increased from 11,000 ± 2,913 at day 0 to 58,900 ± 8,727 at day 22 post-infection. Importantly, significantly higher numbers of CD80^-^PD-L2^-^CD73^+^ B cells (50,200 ± 3,992) were still detected at 1-M PC. Similar results were obtained from a replicate experiment (S1A Fig). Additionally, the number of cells positive for all three markers (CD80^+^PD-L2^+^CD72^+^) was also significantly elevated and peaked at the D-13 time point. CD80^+^PD-L2^+^CD72^+^ B cell frequencies increased from 4,340 +/- 404 at day 0 to 36,200 ± 2,748 at day 13 post-infection (Fig 2C). Replicate data from an independent experiment are shown in S1B Fig (S1B Fig). Dot plots show an increase in CD80^-^PD-L2^-^CD73^+^ B cell frequencies at 1-M PC versus D-0 in naive mice (1.8% vs 6.0%) (Fig 2D). Thus, increased numbers of CD80^-^PD-L2^-^CD73^+^, and CD80^+^PD-L2^+^CD73^+^ B cells were seen at 1-M PC, but the highest frequencies were observed in the CD80^-^PD-L2^-^CD73^+^ B cell population. It should be noted that CD73 expression has been shown to increase during the development of GC B cells [19] and CD73 is a marker for GC-derived IgM^+^ memory B cells [20]. Thus, the CD73^+^ B cell populations we see expanding during the course of malaria infection are likely originating from GC’s.

### PyNL immune B cells expressing CD73 protect against a primary PyNL infection

**Fig 3:**
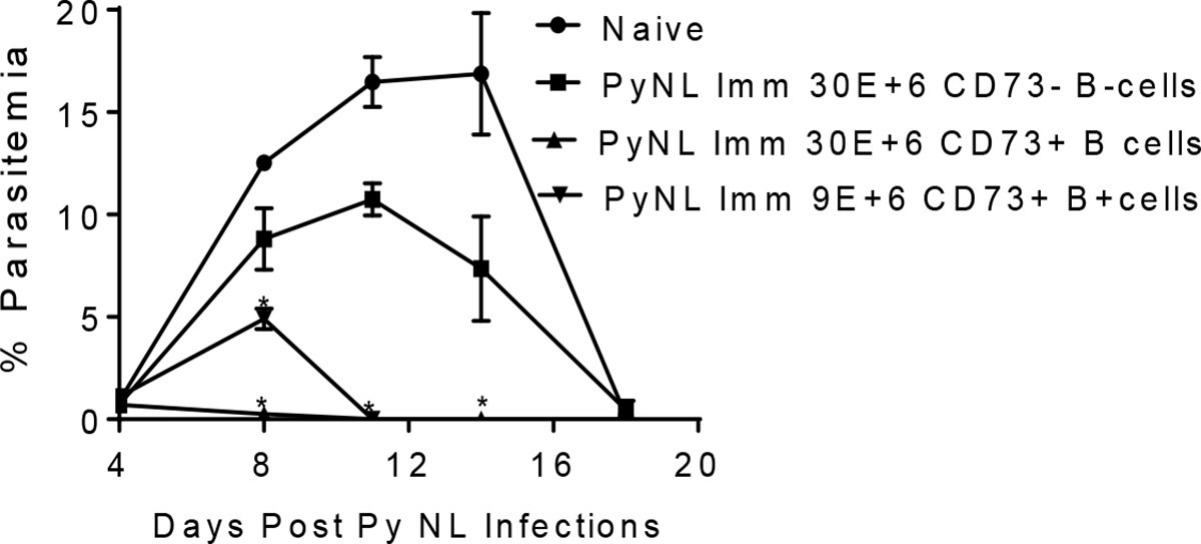
PyNL immune CD73^+^ B cells confer protection against a primary PyNL infection. CD73^-^ (3 x 10^7^) or CD73^+^ enriched B cells (9 x 10^6^ or 3 x 10^7^) obtained from immune mice 7 months post-PyNL clearance were transferred into nonimmune, naive mice. Two hours after the transfer of cells, mice were infected i.p. with 1 x 10^6^ PyNL erythrocytic stage parasites. Parasitemias were evaluated by blood smears starting at day 4 post-infection until parasitemia clearance. Results are expressed as the percent parasitemia ± SEM. A representative experiment out of two experiments is shown. Mann-Whitney test was used for statistical evaluation, p<0.05; n = 5 mice per group.

Since the CD80^-^PD-L2^-^CD73^+^ B cell population was elevated during the primary PyNL infection and persisted at 1-M post-parasitemia clearance, we assessed whether the immune CD73^+^ B cell population could protect against a PyNL infection by adoptive transfer. For these experiments, immune B220^+^CD73^+^ B cells were enriched from spleens at 7 months post-infection using magnetic beads specific for CD73^+^ molecules as described in the Methods section. Following adoptive transfer of 9 x 10^6^ or 3 x 10^7^ B220^+^CD73^+^ cells into nonimmune animals, the mice were infected with 1 x 10^6^ PyNL parasites 1–2 hours later. Concurrently, 3 x 10^7^ immune B220^+^CD73^-^ cells were injected into naive (nonimmune) mice as experimental controls. As seen in Fig 3, parasitemia levels peaked in naive control mice at day 14 (16.8 ± 2.9%) and were cleared at day 20 post-infection. As in naive mice, high parasitemia (10 ± 0.8%) was observed in infected mice injected with 30 million CD73^-^ B cells from immune mice. In contrast, mice that were adoptively transferred with either 9 million or 30 million CD73^+^ B cells had substantially and significantly reduced parasitemias which cleared earlier than the mice in the control groups. Peak parasitemia was reduced from 16.9 ± 2.97% in the naïve mice to 4.9 ± 0.50% in mice receiving 9 million CD73^+^ B cell and to 0.72 ± 0.18% (p = 0.0079) in mice receiving 30 million CD73^+^ B cells. Similar results were obtained for an independent, repeated experiment and are shown in S2 Fig. For this experiment, 9 million CD73^+^ B cells were adoptively transferred into naïve mice which resulted in a post-PyNL challenge reduction in peak parasitemia from 10.7 ± 0.51% to 3.5 ± 0.97% relative to naïve, untreated control mice. These results highlight the protective role of CD73^+^ B cells in PyNL infection.

### PyNL immune CD19^+^ B cells express CD73 and granzyme B (GrB)

**Fig 4:**
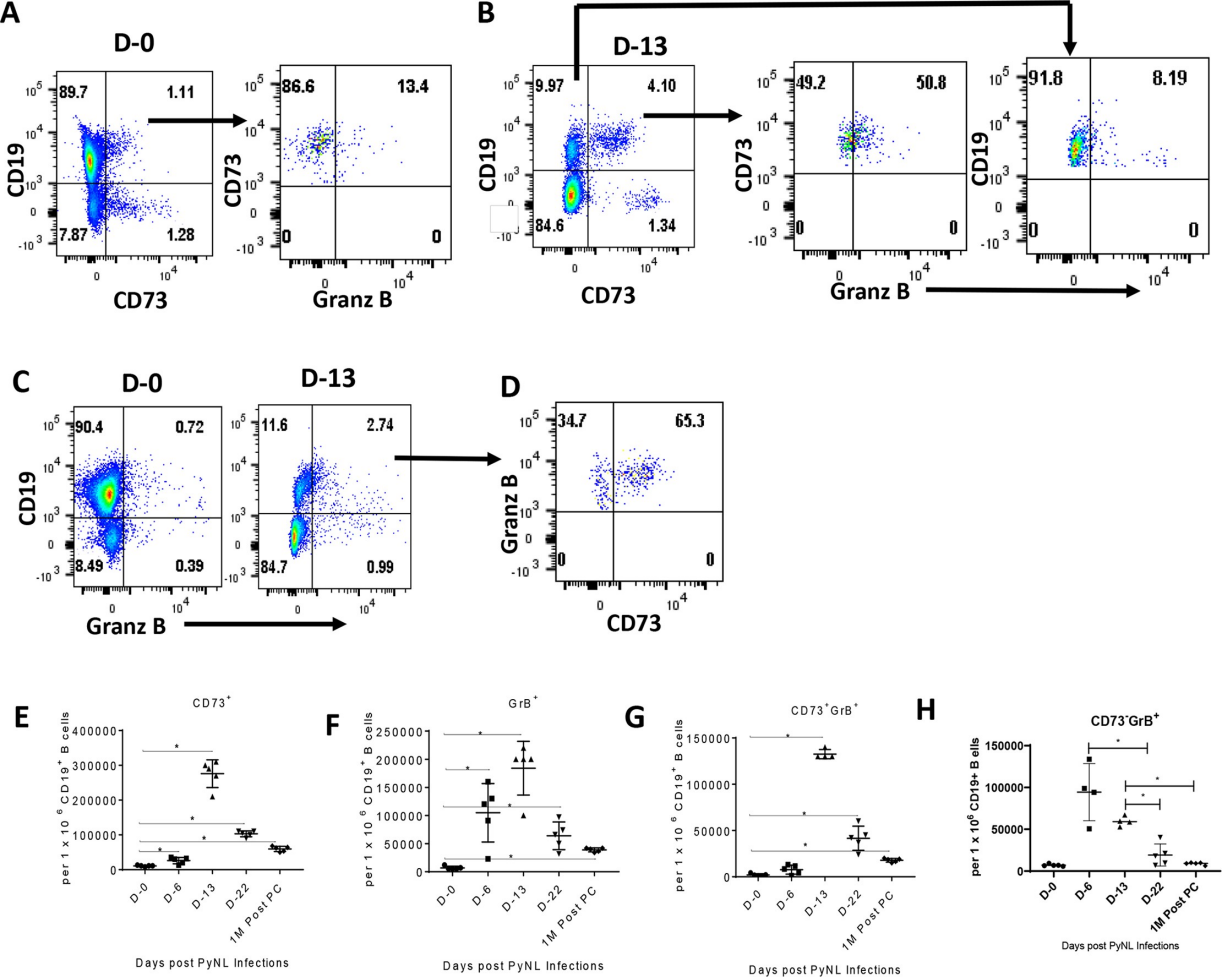
Comparison of CD19^+^ B cell frequencies expressing CD73 and/or Granzyme B at different time points following a primary PyNL infection and at one month post-parasitemia clearance. Representative flow cytometry dot plots show frequencies of CD19^-^ or CD19^+^ splenocytes (T cells were excluded) expressing CD73 or frequencies of CD19^+^CD73^+^ B cells expressing Granzyme B (Granz B) at (A) Day 0 or (B) Day 13 post-PyNL infection. Alternatively, frequencies of CD19^-^ or CD19^+^ splenocytes expressing Granz B at (C) both time points or (D) frequencies of CD19^+^Granz B^+^ cells expressing CD73 at D-13 were determined. Also shown is the development of CD19^+^ B cells expressing (E) CD73, (F) Granzyme B (GrB), (G) both CD73 and GrB or (H) or CD73^-^ B cells expressing GrB following a primary PyNL infection beginning at Day 0 through Day 30 post-parasitemia clearance (PC). A representative experiment of two experiments is shown. Mann Whitney test was used for statistical evaluation. *p<0.05; n = 5 mice per group.

Recently, the induction of B cell-derived granzyme B (GrB) has been described in the context of infectious diseases [21, 22]. In malaria studies, the number of cells expressing GrB has been shown to increase after natural and experimental malaria infections, and in protected, vaccinated subjects following a malaria infection [23]. Since results from our adoptive transfer studies showed that CD73^+^ B cells conferred protection against a PyNL infection, we investigated whether CD19^+^ B cells also expressed GrB. To confirm the kinetics of CD73^+^ B cell formation, CD19^+^ spleen cells were analyzed for CD73 expression by flow cytometry. Fig 4A and 4B contains representative dot plots which show an increase in the percentage of CD19^+^CD73^+^ B cells at D-13 post-infection relative to D-0 (4.1% vs 1.1% respectively). However, when considering only the B cell population, the proportion of CD19^+^ cells staining positive for CD73 was 29% and 1.3% of B cells at D-13 and D-0 respectively. T cells were excluded (gated out) from the analysis. The number of CD19^+^CD73^+^ B cells increased from 10,800 ± 800 at day 0 to 276,000 ± 17,776 at day 13 post-PyNL infection (Fig 4E), and although the numbers decreased thereafter, the CD19^+^CD73^+^ B cell population was still significantly elevated with respect to day 0 of infection at 1-M PC (59,000 ± 3,354) (p<0.05). The proportion of CD19^+^CD73^+^ B cells staining positive for GrB was determined following gating on CD19^+^CD73^+^ cells (Fig 4A and 4B). At D-0, 13% of this population stained positive for GrB while 51% were positive at D-13. Eight percent of CD19^+^CD73^-^ cells also stained positive for GrB at D13 (Fig 4B). In a separate experiment, we also examined the population of CD19^+^GrB^+^ cells expressing the CD73 marker at both time points (T cells were excluded). As shown in Fig 4C, both CD19^+^ and CD19^-^ splenocytes increased production of GrB at D-13 relative to D-0. Consistent with our previous findings (Fig 4A and 4B), GrB expression from CD19^+^ cells increased from 0.7% at D-0 to 2.7% at D-13 and was greatest in the CD19^+^ population. At D-0, 0.8% of CD19^+^ cells expressed GrB which increased to 19% at D-13, (> 30-fold increase). Similar to our previous results, 65% of the CD19^+^GrB^+^ population stained positive for CD73 while 35% were CD19^+^CD73^-^ (Fig 4D). Thus, as shown in Fig 4B and 4D, by Day 13 post-PyNL infection (at peak parasitemia), a substantial fraction (51%) of CD19^+^CD73^+^ cells express GrB and a majority (65%) of CD19^+^GrB^+^ cells express the CD73 marker. Fig 4F and 4G show the number of CD19^+^ splenocytes staining positive for GrB or both CD73 and GrB. The number of CD19^+^GrB^+^ B cells at the peak infection time-point (D-13) was 184,400 ± 21,354 and remained significantly elevated at 1-M PC (38,900 ± 1,584) relative to D-0 (6,860 ± 1,338) (Fig 4F). The number of CD73^+^GrB^+^ B cells at peak infection time point (D-13) was 132,500 ± 2,500 and, at 1-M PC, CD73^+^GrB^+^ B cell frequencies were still significantly elevated relative to D-0 (17,800 ± 902 vs 1,930 ± 609) (p<0.05) (Fig 4G). Thus, CD19^+^CD73^+^GrB^+^ B cells emerged after PyNL infection and their highest frequency overlapped with peak parasitemia (D-13 post-infection). Moreover, although their frequencies decreased after D-13 post-infection, at 1-M post-PyNL infection the CD19^+^CD73^+^GrB^+^ B cell population remained significantly elevated relative to the D-0 time point. GrB production from CD19^+^CD73^-^ cells peaked at D-6 post-infection with a significant increase in the number of cells staining positive for GrB from 7,017 ± 1,401 at D-0 to 94, 331 ± 34,135 at D-6 per 1 x 10^6^ B cells (Fig 4H). By one month post-infection, the number of CD73^-^GrB^+^ cells had declined to 8,898 ± 1,481 per million B cells which was not significantly different from the D-0 time point. Of interest, thus, is the observation that GrB production by B cells preceded CD73 expression suggesting the possibility that a subset of CD73^+^ B cells originated from the GrB^+^ B cell population.

### PyNL immune CD19^+^ B cells expressing CD73 and GrB also express IgM and were expanded at day 6 post-secondary PyNL infection

**Fig 5:**
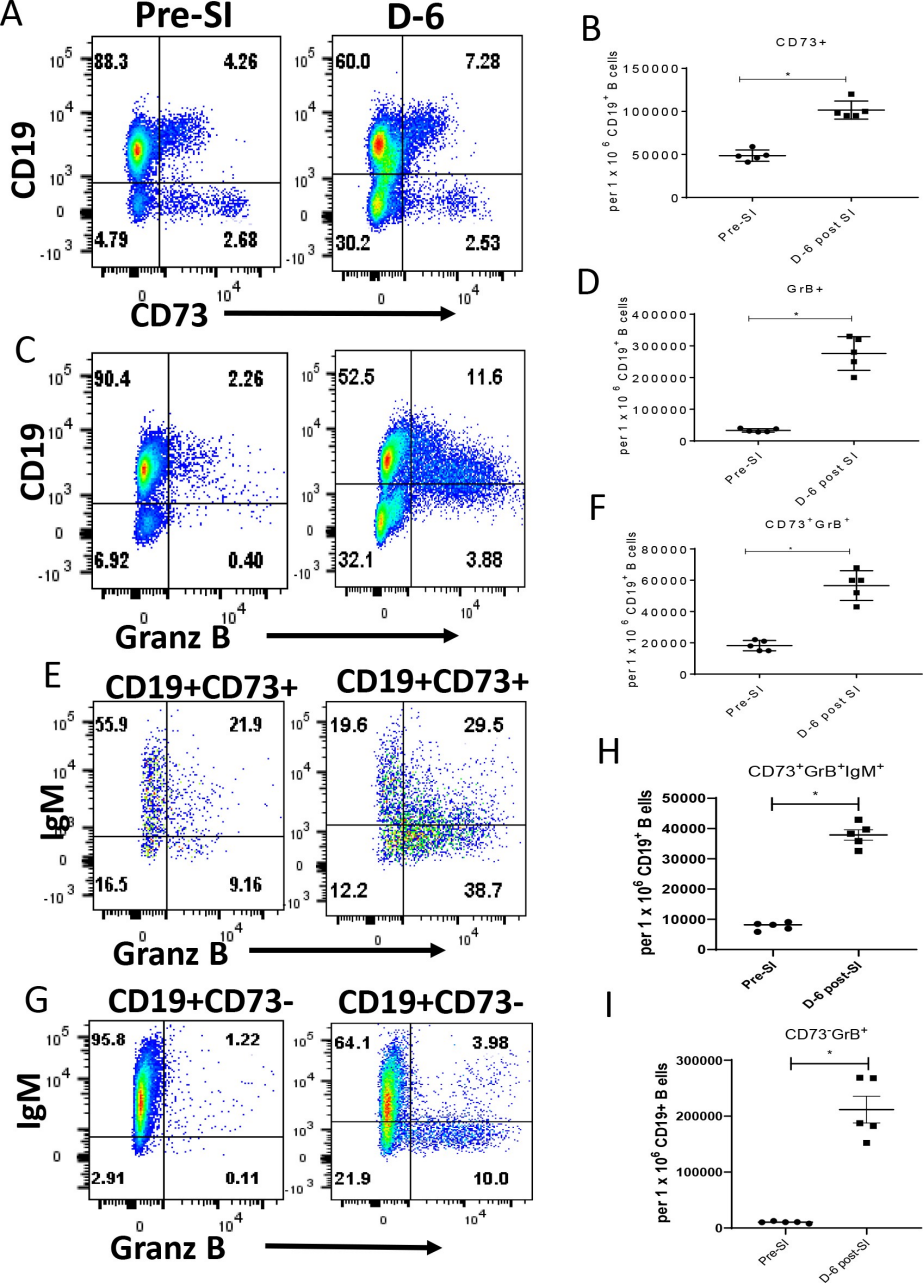
Comparison of CD19+ B cell frequencies expressing CD73 and GrB the day before and at Day 6 post-secondary infection with PyNL. Splenic B cells were recovered from PyNL immune mice two months post-primary PyNL infection (Pre-SI) or six days post-secondary PyNL infection (D-6 post-SI) (secondary infection at 2 months post-primary infection). Frequencies of CD19^+^ B cells expressing (A, B) CD73, (C, D) GrB, (E, F) CD73 and GrB, (E, H) CD73, GrB, and IgM markers or (G, I) CD19^+^CD73^-^, GrB^+^, IgM^+^ cells were measured by flow cytometry at these time points. Representative dot plots (A, C, E, G) show frequencies of these different populations at Pre-SI and D-6 post-SI. Representative experiment of two experiments is shown. Mann-Whitney test was used for statistical evaluation. Mean ± SEM; *p<0.05; n = 5 mice per group.

Since CD19^+^CD73^+^GrB^+^ B cell numbers were still elevated at 1-M PC, we wanted to determine if these cells expanded after a second PyNL infection (D-6 post-secondary infection) relative to the day before a second PyNL infection (Pre-SI). We selected day 6 because at this post-infection time point parasitemia begins to decline in PyNL immune mice and in mice receiving enriched CD73^+^ B cells (Figs 1 and 3). Examining splenocytes by flow cytometry, the frequencies (%) of CD19^+^CD73^+^ (7.3% vs 4.3%), CD19^+^GrB^+^ (11.6% vs 2.3%), and CD73^+^GrB^+^ (68% vs 31% of the CD73^+^ population) B cells were higher at D-6 post-SI relative to Pre-SI (Fig 5A, 5C and 5E, respectively). The frequency of CD73^-^ B cells expressing GrB was 1.33 and 14% at Pre-SI and D-6 respectively (Fig 5G). Additionally, by gating on GrB^+^ B cells, we determined the frequencies of CD19^+^GrB^+^ cells expressing the CD73 marker (66%) or without the marker (CD19^+^GrB^+^CD73^-^) (34%) (S3A Fig). Correspondingly, the number of CD19^+^CD73^+^, CD19^+^GrB^+^, CD73^+^GrB^+^ and CD73^-^GrB^+^ B cells were also higher at D-6 post SI compared to Pre-SI (Fig 5B, 5D and 5F). The CD19^+^CD73^+^ population increased from 48,600 ± 2,943 at Pre-SI to 101,600 ± 4,697 at D-6 post-SI; the CD19^+^GrB^+^ population increased from 33,200 ± 2,437 at Pre-SI to 276,600 ± 23,791 at D-6 post-SI, and the CD73^+^GrB^+^ population increased from 18,200 ± 1,463 at Pre-SI to 56,600 ± 4,238 D-6 post-SI. Likewise, the number of CD73^-^GrB^+^ B cells increased from 10,318 ± 1,714 at Pre-SI to 211,742 ± 53,389 at D-6 (Fig 5I). Similarly, after gating on CD19^+^GrB^+^ cells, the number of GrB-expressing B cells also expressing CD73 was found to increase at D-6 post-secondary infection with 16,314 ± 4,067 expressing the CD73 marker at Pre-SI and 44,828 ± 8,719 at D-6, and 12,774 ± 2,162 vs. 135,144 ± 38,099 for CD19^+^GrB^+^CD73^-^ B cells at Pre-SI and D-6 respectively (S3B Fig). Of note, the number of CD19^+^CD73^+^IL10^+^ B cells and CD19^+^CD73^+^CD159^+^ B cells were less than 700 per 1 x 10^6^ CD19^+^ B cells at D-6 post-SI, indicating the absence of B reg and NK cells (data not shown).

Since IgM^+^ B cells have recently been shown to rapidly respond to malaria re-infection [18], we next examined whether PyNL CD19^+^CD73^+^GrB^+^ B cells also expressed the IgM marker by first gating on these triple positive cells. Fig 5E and 5H show an increase in the frequency of CD19^+^CD73^+^GrB^+^IgM^+^ B cells from 22% to 30% (7,286 ± 494 to 37,026 ± 1,252) at Pre-SI and D-6 post-SI respectively. Thus, these results show that CD19^+^CD73^+^GrB^+^ B cells can also express IgM^+^ and are expanded soon after a second PyNL infection. The number of CD73^-^ B cells expressing both IgM and GrB was found to be 8,859 ± 960 and 52,439 ± 16,402 at Pre-SI and D-6 respectively (not shown), and the percentages were 1.22 and 4.0% respectively (Fig 5G). We also compared the frequencies of CD19^+^IgM^+^ cells expressing CD73 and/or GrB by first gating on CD19^+^IgM^+^ double positive cells. The mean percentages (±SEM) of CD19^+^IgM^+^ cells staining positive for CD73 (4.3 ± 0.28 vs. 8 ± 0.2), GrB (2.8 ± 0.5 vs. 20 ± 5.7) or both CD73 and GrB (1.2 ± 0.4 vs. 4.7 ± 1.0) at Pre-SI vs. D-6 post-SI respectively are shown in S4 Fig.

### *In vitro* recall response

**Fig 6:**
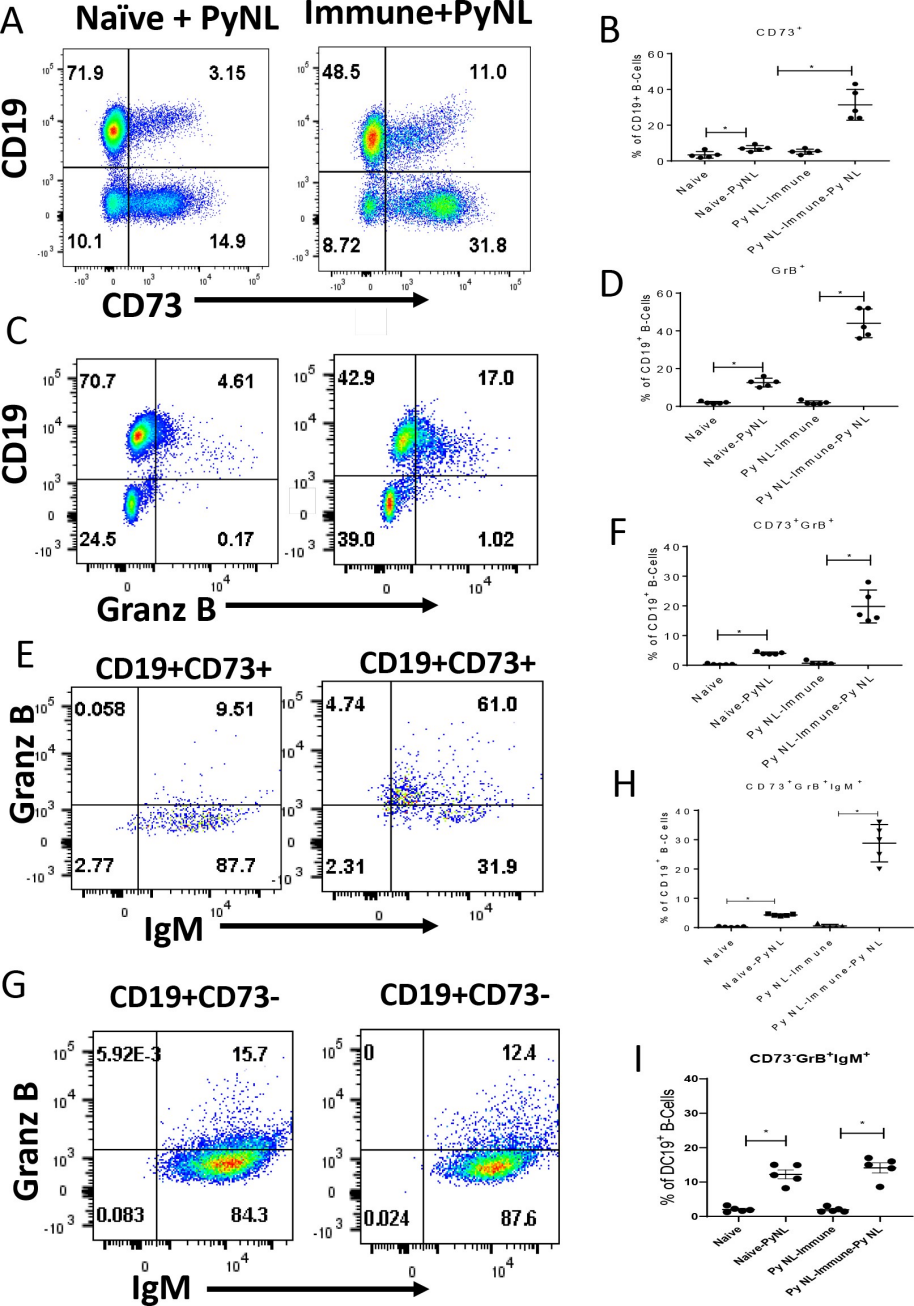
Comparison by flow cytometry of CD19^+^ B cell frequencies expressing CD73, IgM or Granzyme B (GrB) following an *in vitro* PyNL infection. Splenic CD19^+^ B cells were recovered from noninfected mice (naive) or at 4 months post-PyNL clearance (PyNL Immune mice). Five million spleen cells were co-cultured with 5 x 10^6^ PyNL iRBC (Naive-PyNL; PyNL Immune-PyNL) or without PyNL iRBC (Naive; PyNL Immune). Representative flow cytometry dot plots from two independent experiments show frequencies of different B cell subsets from stimulated Naïve (left column) or Immune splenocytes (right column) (A, C), or after gating on CD19^+^CD73^+^ (E), or CD19^+^CD73^-^ cells (G). Frequencies of B cell subsets shown in B, D, F, H and I are expressed as the percentage of CD19^+^ cells and, thus, were obtained after gating on CD19^+^ B cells as shown on the y-axis (n = 5 mice per group). Mann-Whitney test was used for statistical evaluation. *p<0.05; n = 5 mice per group.

Next, we sought to determine the *in vitro* recall response of these B cell subsets in PyNL experienced mice. For this study, we measured the frequencies of B cell subsets following the stimulation of splenocytes with PyNL infected RBCs (iRBCs) for 4 days from naive mice and from mice that had recovered from a primary PyNL infection 4 months earlier. Representative dot plot data are shown for splenocytes derived from Naïve (left column) or PyNL-Immune (right column) mice after incubation with iRBCs (Fig 6A, 6C, 6E and 6G). Following *in vitro* stimulation, we found a significant increase in the frequencies of splenic CD19^+^ B cells expressing CD73 (Fig 6A) (from 3.15 to 11%), or GrB (Fig 6C) (from 4.6 to 17%) derived from Naïve and Immune mice respectively. After gating on CD19^+^CD73^+^ cells, the frequency of cells in this population expressing GrB was found to increase from 9.5 to 65.7% (Fig 6E) and the proportion expressing IgM was 97.5% in the stimulated Naïve group and 93% in the stimulated Immune group (Fig 6E). The proportion of CD73^+^GrB^+^ B cells expressing IgM increased from 9.5% to 61% in the Naïve and Immune groups respectively (Fig 6E). The frequency of the CD73^-^ B cell population expressing GrB was not appreciably different between stimulated Naïve and Immune splenocytes (16 vs. 12%), and the frequency expressing IgM remained the same at around 98% for stimulated Naïve and Immune splenocytes (Fig 6G).

The mean frequency (%) (± SEM) of the different B cell subsets in unstimulated Naïve, stimulated Naive (Naïve-PyNL), unstimulated Immune (PyNL-Immune) and stimulated Immune (PyNL-Immune-PyNL) groups (n = 5 mice per group) were each obtained after gating on CD19^+^ cells (as shown on the y-axis) and are shown in Fig 6B, 6D, 6F, 6H and 6I. The frequency of CD19^+^ B cells expressing CD73 (Fig 6B) or GrB (Fig 6D) was significantly elevated in the stimulated PyNL-Immune group (PyNL-Immune-PyNL) relative to the other groups with at least a 4.5 or 3.5-fold increase respectively. The proportion of CD19^+^ cells expressing both CD73 and GrB was also significantly elevated in the PyNL-Immune-PyNL group with at least a 6-fold increase observed relative to the control groups (Fig 6F). As shown in Fig 6H, the percentage of CD19^+^ cells expressing CD73, GrB and IgM (CD19^+^CD73^+^IgM^+^GrB^+^) was significantly elevated in the stimulated, Immune group relative to the control groups with a ≥ 6-fold increase. Fig 6I shows that the frequencies of CD73^-^ B cells expressing GrB and IgM were not different between the Naïve and PyNL stimulated groups, and both were significantly elevated relative to their unstimulated counterparts (6–7 fold increase). Although there was a significant increase in the frequencies of all the B cell subsets among the naïve groups (stimulated versus unstimulated naïve splenocytes) (minus the CD73^+^IgM^+^ group), the difference between these two groups remained markedly lower than the differences between stimulated PyNL immune splenocytes and the other control groups (from Fig 6B, 6D, 6F, 6H and 6I).

**Fig 7:**
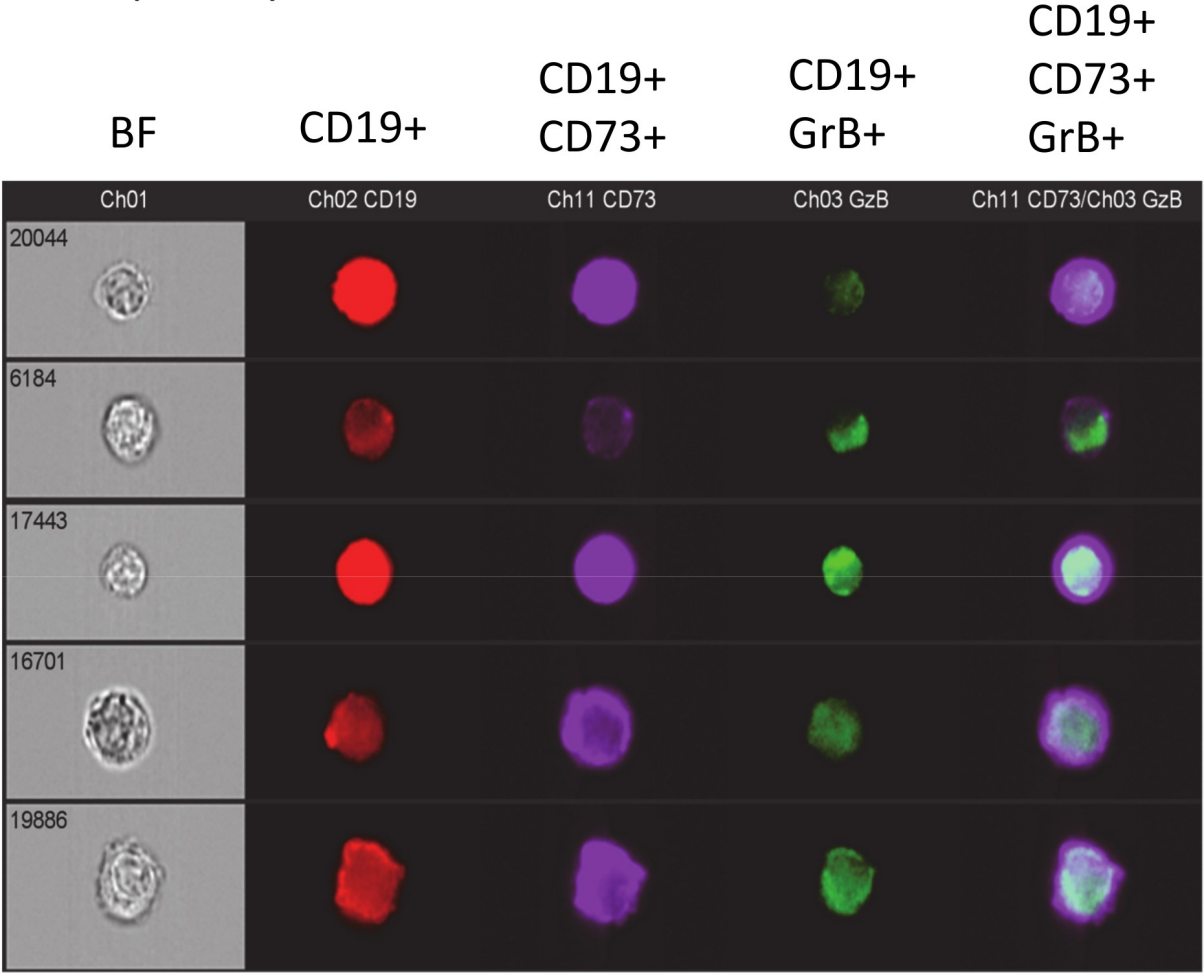
Flow Cytometry Imaging of CD73^+^GrB^+^ B cells. Splenocytes from PyNL immune mice were co-incubated with PyNL infected erythrocytes for 4 days and then surface stained with fluorochrome-conjugated antibodies specific for CD19, CD73, and CD8 followed by intracellular staining using a monoclonal antibody specific for granzyme B (GrB). The cells were incubated with violet live-dead stain to differentiate viable from nonviable cells and then visualized using an ImageStream system via bright field (BF) or fluorescence mode at 60X magnification (CD8^+^ cells were excluded from the gates). Using fluorescence mode, CD73^+^ B cells expressing GrB were visualized by placing a gate on live CD19^+^ cells (red) followed by gating on CD73^+^ (purple), GrB^+^ (green) or CD19^+^CD73^+^GrB^+^ cells (purple and green).

The expression of CD73 and GrB by CD19^+^ cells was visually confirmed using ImageStream analysis. Representative images are shown in Fig 7. Importantly, the fold differences in the frequencies of CD19^+^ cells expressing both CD73 and GrB between naïve and PyNL-immune parasite-stimulated splenocytes as determined using the ImageStream system, were similar to that observed using conventional flow cytometry (Fig 6F). For parasite-stimulated immune and naïve (not shown) splenocytes, 12 ± 1.2% and 1.1 ± 0.35% (p < 0.0001) respectively of the CD19^+^ B cells were CD73^+^GrB^+^. In contrast, as a control, the frequency of CD73^+^GrB^+^ B cells from PyNL-immune splenocytes incubated without parasites was 2.9 ± 0.24% which was significantly lower than that observed for PyNL-stimulated immune B cells (p < 0.0001) (not shown). Overall, flow cytometry data show the marked expansion of the CD19^+^CD73^+^, CD19^+^GrB^+^, and CD19^+^CD73^+^GrB^+^ B cell subsets from PyNL experienced mice in response to PyNL iRBCs, underscore their memory phenotype and suggest their relevance in protection against re-infection with malaria parasites.

Finally, to assess whether the increased recall response in PyNL immune mice following PyNL iRBCs stimulation also resulted in the release of GrB, we measured GrB levels in the supernatants of the *in vitro* stimulated splenocytes (S5 Fig). Following a 4 day incubation period, only the supernatants of PyNL immune splenocytes co-cultured with PyNL iRBCs (PyNL Immune-PyNL) contained significantly higher amounts of GrB compare to the unstimulated PyNL immune (PyNL Immune) and the stimulated naïve control groups (Naïve-PyNL) (p = 0.0267 and p = 0.0038 respectively). Thus, GrB production and secretion can be augmented after a second exposure to PyNL parasites.

## Discussion

Despite the considerable public health importance of malarial disease, mechanisms of immune protection against malaria are not well understood [24, 25]. To investigate anti-malarial immunity, we evaluated the involvement of B cell subsets in the murine PyNL re-infection model. Our overall findings were consistent with earlier results [5, 16]. Mice given a primary PyNL infection acquired a prolonged state of nearly sterile immunity which persisted for at least 9 months.

Frequently, a successful anti-pathogen immune response is dependent on the development of long-lived plasma cells and MBC. However, the complexity of MBC development and the heterogeneity of the MBC subsets generated have impeded our understanding of the origins and functions of these cells. In mice, availability of a limited number of MBC markers and uncertainties about the role of the diverse MBC subsets have hindered the study of murine MBC immunity. Using transgenic mice that bear a single BCR in response to a single T-cell dependent antigen (nitro-phenyl), Shlomchik and colleagues reported that memory B cell subsets can express PD-L2, CD80 and CD73 as markers [12, 13, 17]. These B cell subsets were characterized as diverse and heterogenous populations that were biologically distinct from each other. The independence of these MBC subsets was not defined by class switching because IgM^+^ and IgG^+^ cells were identified in each subset. Although these three B cell markers were useful for characterizing memory B cells induced by a single well-defined antigen, it has been unclear whether these markers would be relevant for identifying MBC subsets specific to other types of antigens. Recently, Bemark *et al*. showed that functional MBC can be identified after adoptive transfer of CD80^+^ (but not CD80^-^) B cells at 1 year post-immunization with a cholera toxin adjuvanted antigen preparation [26]. Additionally, Onodera *et al*. demonstrated that CD73, CD80, and PD-L2 expressing MBC generated in response to an influenza infection persisted for at least 5 months and, after re-infection, differentiated into plasma cells that produced neutralizing antibodies [15].

In our studies, we found that CD73^+^ B cells transferred from mice 4 months after their recovery from PyNL infection conferred protection against a primary PyNL infection in naive mice. The presence of CD73 molecules on protective B cell subsets is somewhat puzzling because CD73 is expressed in response to inflammation and can suppress pro-inflammatory cytokine production [14]. Expression of CD73 molecules have been associated with limiting microbial spread during sepsis, reduced inflammatory cytokine levels, and reduced injury to organs. In some infection models, CD73 actually impairs immunity by attenuating inflammation [27]. However, further analysis of the PyNL immune CD73^+^ B cell population suggests possible biological mechanisms unrelated to inflammation by which these cells could limit malarial infections. For example, the presence of surface IgM on most of the immune CD73^+^ splenocytes indicates that these cells resemble IgM^+^ memory B cells recently described in a *P*. *chabaudi* malaria study [18]. These *P*. *chabaudi* immune IgM^+^ B cells were shown to be high affinity rapid responders that initiated an apparent early antibody response to a secondary infection. It is probable that the PyNL immune IgM^+^CD73^+^ B cells could constitute the majority of early responding antibody-secreting plasmablasts.

In our *in vivo* studies, we found that some CD73^+^ B cells also expressed GrB. This atypical B cell sub-population was significantly increased at day 13 post-infection compared to day zero during the primary PyNL infection and were still elevated at 1 month post-parasitemia clearance. When mice were re-infected with PyNL parasites, an increase in CD73^+^, GrB^+^, CD73^+^GrB^+^, and CD73^+^GrB^+^IgM^+^ B cell frequencies were observed at day 6 post-infection compared to frequencies seen the day before infection. A similar increase in CD73^+^, GrB^+^, CD73^+^GrB^+^ and CD73^+^GrB^+^IgM^+^ B cells were observed at day 4 post-PyNL *in vitro* infection. The CD73^+^GrB^+^ B cells did not express IL-10 or CD159, indicating the lack of contamination with NK and/or B reg cells.

Notably, CD19^+^CD73^-^ B cells were also protective against a primary PyNL infection, but not as protective as CD73^+^ B cells, and, interestingly, CD73^-^ B cells also expressed GrB. The percentage of these cells was more than five-fold less than CD73^+^ cells expressing GrB at day 13 at peak parasitemia, peaking a week earlier at day 6. Furthermore, the number (per million B cells) of CD73^-^GrB^+^ B cells at day 6 post-primary infection was almost as high as the peak number of CD73^+^GrB^+^ at day 13, but declined rapidly thereafter. As shown in Fig 4E and 4F, the expression of GrB precedes (day 6) the expression of CD73 (day 13) from the B cell population following a primary infection. Thus, the kinetic data shown in this figure suggests that CD73^+^GrB^+^ cells are likely originating from CD73^-^GrB^+^ B cells a week after the onset of GrB expression. Two months following a primary infection, the number of CD73^+^GrB^+^ cells was two-fold greater than CD73^-^GrB^+^ B cells, but by day 6 post-secondary infection, the number greatly expanded to more than three-fold the number of CD73^+^GrB^+^ cells. If GrB expression is involved in protection, B cell subsets in the CD73^-^ population (possibly CD80^+^ cells) may be playing a greater protective role than CD73^+^ cells post-secondary PyNL infection, or GrB expression is preceding the expression of CD73 from the CD73^-^ population post-secondary challenge as well. Interestingly, the frequency of CD73^-^GrB^+^ cells did not differ between naïve and PyNL experienced mice after stimulation indicating these cells may not be capable of recognizing antigen *in vitro*. Future work will focus on delineating the protective role of GrB and more fully characterizing protective B cells against a PyNL primary and secondary infection.

We also found an increase in GrB levels in PyNL immune splenocyte culture supernatants following incubation with PyNL parasites. However, we cannot be certain that all the measured GrB was produced by B cells since other mouse splenocytes can also produce GrB. Our finding of an increase in GrB^+^ B cells following PyNL infection is novel. B cell-derived GrB has recently been described in the context of autoimmune diseases such as systemic lupus erythematosus and infectious diseases including mononucleosis and SIV infection [21, 22, 28]. Atypical memory B cells in the context of malaria infection in humans have also been previously reported by others. Weiss et al. described an atypical CD19^+^CD21^-^CD27^-^CD10^-^ hyporesponsive MBC subset in *P*. *falciparum* infected adults and children [29]. This subset exhibited reduced B cell receptor signaling and effector functions (similar to exhausted MBC’s), expressed T-bet and was most prominent in children with chronic asymptomatic malaria infections [30]. A protective role for these atypical MBC’s was posited in that these cells may be anti-inflammatory and thereby function to protect against clinical disease.

Of relevance to our paper is the emerging role of GrB in the immune response to malaria infections. Increased concentrations of systemic GrB have been observed during natural and experimental malaria infections [23]. Elevated numbers of CD8^+^GrB^+^ T cells have also been reported in humans that were protected against *P*. *falciparum* malaria after being immunized with *P*. *falciparum* sporozoites while undergoing chloroquine prophylaxis [31]. Furthermore, *in vitro* experiments have shown that purified GrB has anti-malarial activity against *P*. *falciparum* parasites [32]. Our findings suggest that GrB produced by B cells in PyNL experienced mice may have a critical role in host resistance to re-infection.

Taken together, using a PyNL infection model, we have identified a sub-set of CD73^+^ B cells that express GrB and IgM, have memory-like characteristics and protect against a second PyNL infection. These findings expand our understanding of the role of B cell immunity in response to PyNL infection. The possible protective role of B cell-derived GrB in immunity to malaria is intriguing and merits further investigation.

## Supporting information

S1 Fig CD80^-^PDL2^-^CD73^+^ and CD80^+^PDL2^+^CD73^+^ B cells expand post-primary infection. The number (mean ± SEM) of splenic B cell subsets per 10^6^ total CD19^+^ cells were determined by flow cytometry before (D-0) and up to 1 or 2 months post-PyNL clearance (PC). Mann-Whitney test was used for statistical evaluation; *p<0.05; n = 5 mice per group. Representative experiment of two experiments.
(TIF)

S2 Fig PyNL immune CD73^+^ B cells confer protection against a primary PyNL infection. Nine million CD73^+^ B cells obtained from immune mice 7 months post-PyNL clearance were transferred into nonimmune mice and were infected i.p. two hours after the adoptive transfer with 1 x 10^6^ PyNL erythrocytic stage parasites. Parasitemias were evaluated by blood smears starting at day 6 post-infection until parasitemia clearance. Results are expressed as the percent parasitemia ± SEM. Mann-Whitney test was used for statistical evaluation, p<0.05; n = 5 mice per group.
(TIF)

S3 Fig Comparison of CD19^+^GrB^+^ cell frequencies expressing CD73 and IgM the day before and at Day 6 post-secondary infection with PyNL. Splenic B cells were recovered from PyNL immune mice two months post-primary PyNL infection (Pre-SI) or six days post-secondary PyNL infection (D-6 post-SI) (secondary infection at 2 months post-primary infection). Frequencies of CD19^+^GrB^+^ cells expressing CD73 +/- IgM or CD19^+^GrB^+^CD73^-^ +/- IgM at these time points were measured by flow cytometry. (A) Representative dot plots showing frequencies of these populations at Pre-SI and D-6 post-SI, and (B) the number of CD73^+^ or CD73^-^ B cells per million CD19^+^GrB^+^ cells at these time points (n = 5). Representative experiment of two experiments is shown. Mann-Whitney test was used for statistical evaluation. Mean ± SEM; *p<0.05; n = 5 mice per group.
(TIF)

S4 Fig IgM^+^ B cells expressing CD73 and GrB expand post-secondary infection with PyNL parasites. To determine the extent of expansion of IgM^+^ B cells expressing CD73 and/or GrB, splenocytes were surface and intracellularly stained with specific antibodies before (Pre-SI) or 6 days following a secondary PyNL infection and then analyzed by flow cytometry. After gating on CD19^+^IgM^+^ B cells, the frequency of this population expressing CD73 and/or GrB was determined. One-way ANOVA and unpaired t-test were used to test for statistical significance. Asterisks indicate significant differences relative to corresponding B cell subset frequencies at the Pre-SI time point (Mean ± SEM; p < 0.001). n = 5 mice.
(TIF)

S5 Fig PyNL iRBCs induce GrB production *in vitro* from PyNL immune splenocytes. Splenocytes were recovered from PyNL immune mice (n = 3) 4 months post-parasitemia clearance (PyNL Immune) or from naive (noninfected) mice. 5 x 10^6^ spleen cells from individual mice were co-cultured in the presence or absence of 5 x 10^6^ PyNL- iRBCs. After a 4 day incubation *ex vivo*, supernatants were harvested and GrB was measured in the supernatants of cultures containing naive spleen cells co-cultured with PyNL parasites (Naïve-PyNL), PyNL immune spleen cells alone (PyNL Immune), or PyNL immune spleen cells co-cultured with PyNL parasites (PyNL Immune-PyNL). Mann-Whitney test was used for statistical evaluation. *Statistical significance of PyNL immune-PyNL relative to PyNL Immune and Naïve-PyNL; p = 0.0267 and 0.038 respectively. One of two experiments with similar results is shown.
(TIF)
