# Supplementary figures and images for: Memory CD73+IgM+ B cells protect against Plasmodium yoelii infection and express Granzyme B
Source: PLoS One. 2020 Sep 4;15(9):e0238493. doi: 10.1371/journal.pone.0238493 (PMC7473529; doi:10.1371/journal.pone.0238493)

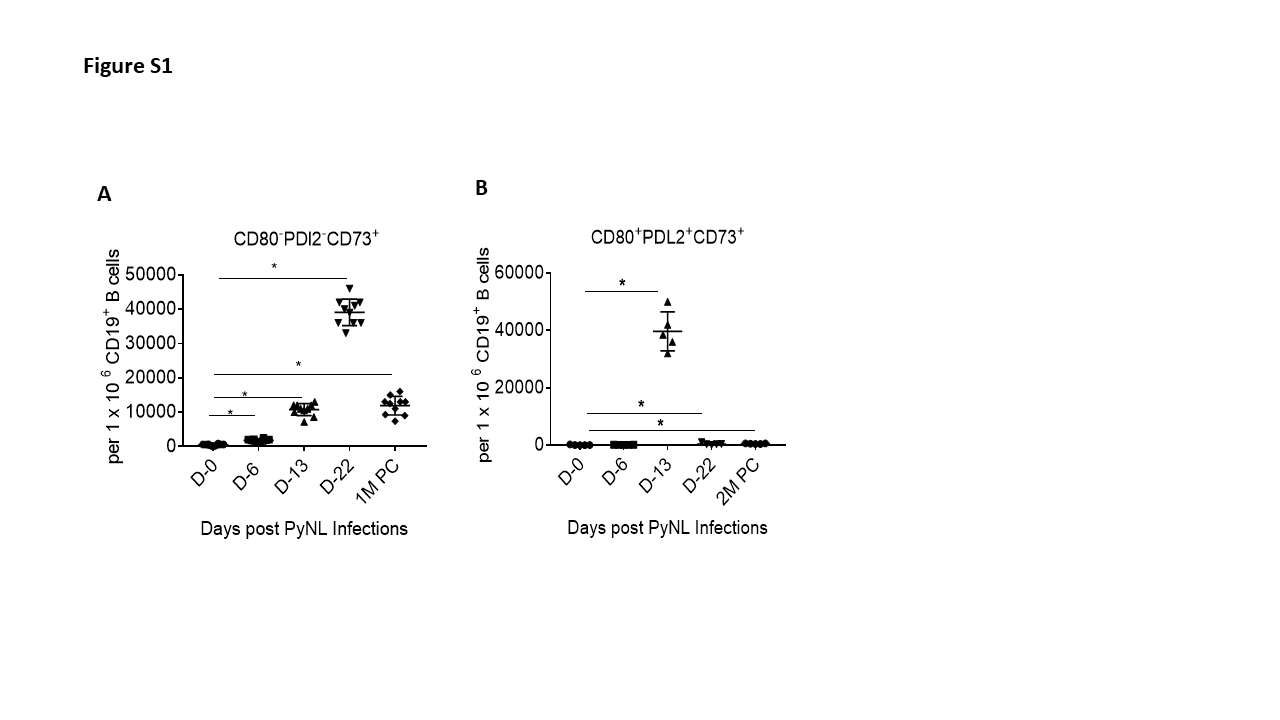

Supplement: S1 Fig — The number (mean ± SEM) of splenic B cell subsets per 106 total CD19+ cells were determined by flow cytometry before (D-0) and up to 1 or 2 months post-PyNL clearance (PC). Mann-Whitney test was used for statistical evaluation; *p<0.05; n = 5 mice per group. Representative experiment of two experiments. (TIF) [file pone.0238493.s001.tif]

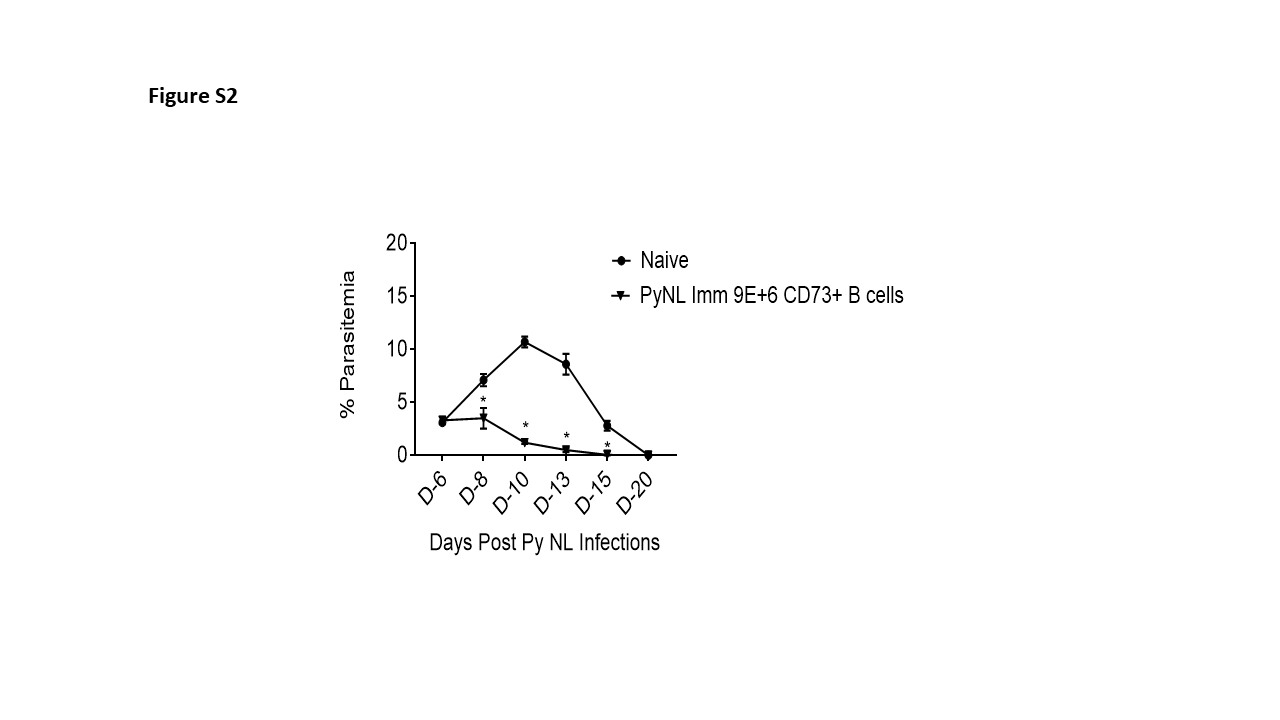

Supplement: S2 Fig — Nine million CD73+ B cells obtained from immune mice 7 months post-PyNL clearance were transferred into nonimmune mice and were infected i.p. two hours after the adoptive transfer with 1 x 10^6^ PyNL erythrocytic stage parasites. Parasitemias were evaluated by blood smears starting at day 6 post-infection until parasitemia clearance. Results are expressed as the percent parasitemia ± SEM. Mann-Whitney test was used for statistical evaluation, p<0.05; n = 5 mice per group. (TIF) [file pone.0238493.s002.tif]

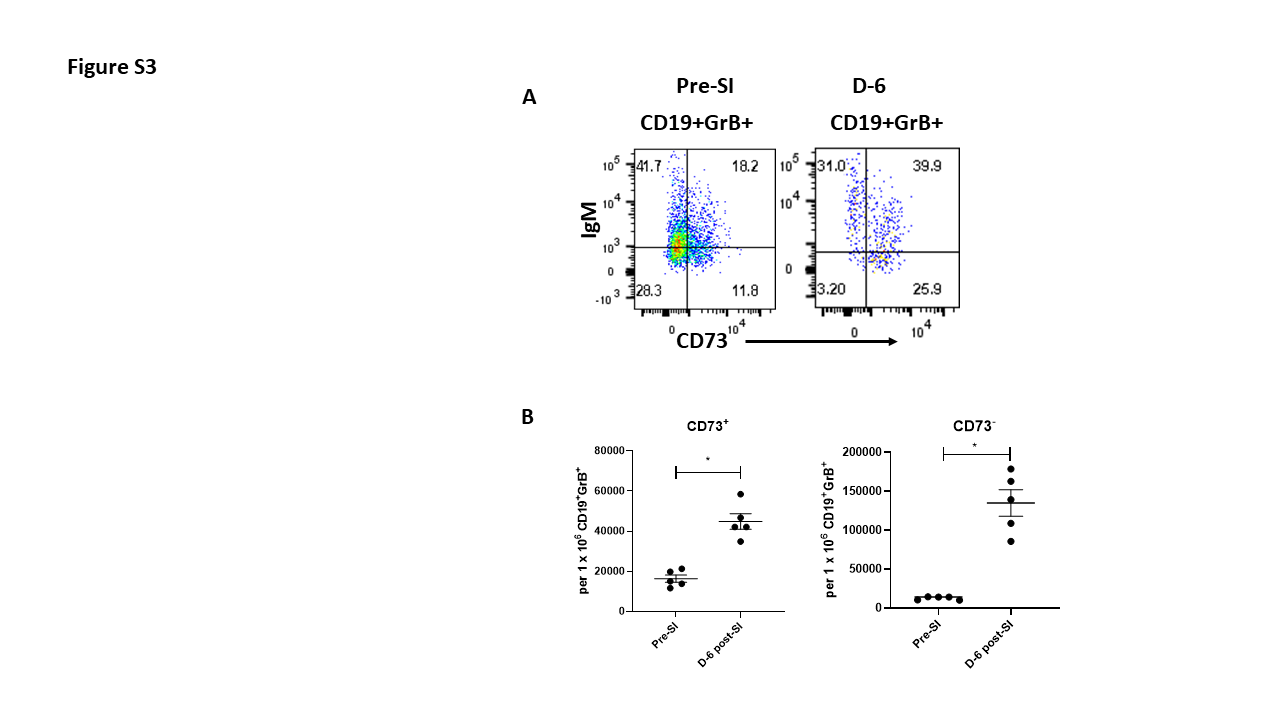

Supplement: S3 Fig — Splenic B cells were recovered from PyNL immune mice two months post-primary PyNL infection (Pre-SI) or six days post-secondary PyNL infection (D-6 post-SI) (secondary infection at 2 months post-primary infection). Frequencies of CD19+GrB+ cells expressing CD73 +/- IgM or CD19+GrB+CD73- +/- IgM at these time points were measured by flow cytometry. (A) Representative dot plots showing frequencies of these populations at Pre-SI and D-6 post-SI, and (B) the number of CD73+ or CD73- B cells per million CD19+GrB+ cells at these time points (n = 5). Representative experiment of two experiments is shown. Mann-Whitney test was used for statistical evaluation. Mean ± SEM; *p<0.05; n = 5 mice per group. (TIF) [file pone.0238493.s003.tif]

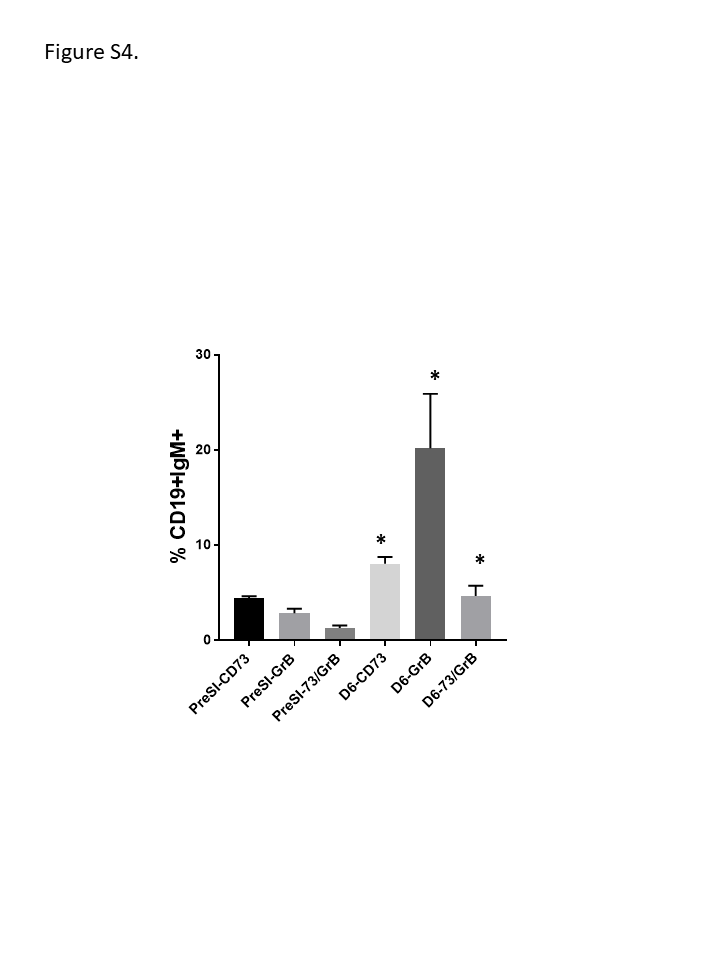

Supplement: S4 Fig — To determine the extent of expansion of IgM+ B cells expressing CD73 and/or GrB, splenocytes were surface and intracellularly stained with specific antibodies before (Pre-SI) or 6 days following a secondary PyNL infection and then analyzed by flow cytometry. After gating on CD19+IgM+ B cells, the frequency of this population expressing CD73 and/or GrB was determined. One-way ANOVA and unpaired t-test were used to test for statistical significance. Asterisks indicate significant differences relative to corresponding B cell subset frequencies at the Pre-SI time point (Mean ± SEM; p < 0.001). n = 5 mice. (TIF) [file pone.0238493.s004.tif]

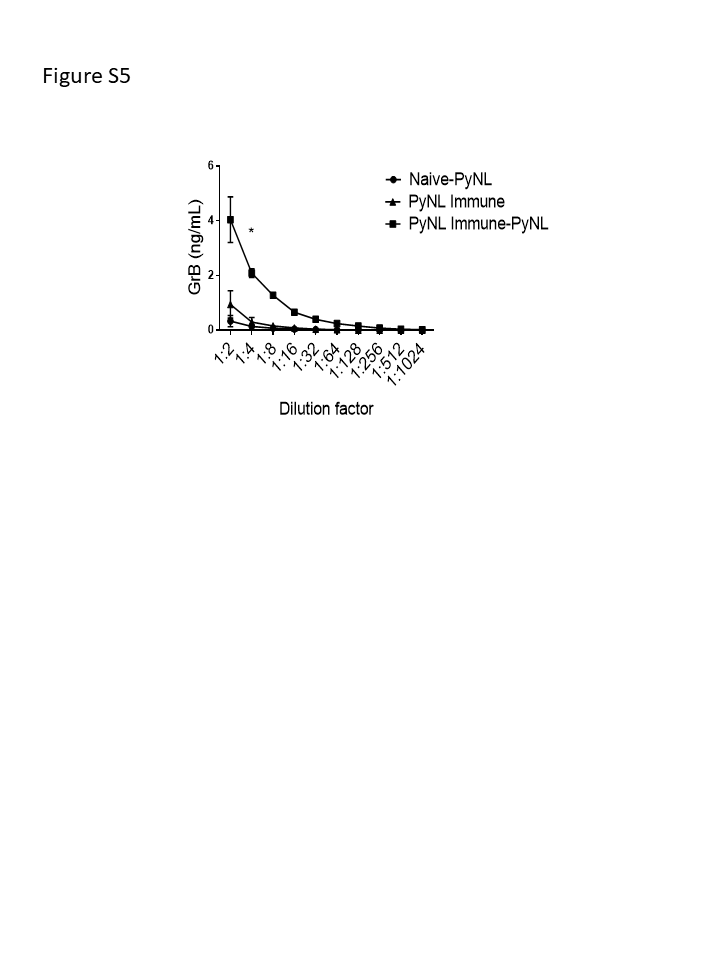

Supplement: S5 Fig — Splenocytes were recovered from PyNL immune mice (n = 3) 4 months post-parasitemia clearance (PyNL Immune) or from naive (noninfected) mice. 5 x 10^6^ spleen cells from individual mice were co-cultured in the presence or absence of 5 x 10^6^ PyNL- iRBCs. After a 4 day incubation ex vivo, supernatants were harvested and GrB was measured in the supernatants of cultures containing naive spleen cells co-cultured with PyNL parasites (Naïve-PyNL), PyNL immune spleen cells alone (PyNL Immune), or PyNL immune spleen cells co-cultured with PyNL parasites (PyNL Immune-PyNL). Mann-Whitney test was used for statistical evaluation. *Statistical significance of PyNL immune-PyNL relative to PyNL Immune and Naïve-PyNL; p = 0.0267 and 0.038 respectively. One of two experiments with similar results is shown. (TIF) [file pone.0238493.s005.tif]
